# Computer physician order entry (CPOE) as a strategy to estimate laboratory activity and costs associated with cancer clinical trials

**DOI:** 10.11613/BM.2018.030706

**Published:** 2018-10-15

**Authors:** Enrique Rodriguez-Borja, Africa Corchon-Peyrallo, Macarena Diaz-Gimenez, Arturo Carratala-Calvo

**Affiliations:** Laboratory of Biochemistry, Valencia University Clinic Hospital, Valencia, Spain

**Keywords:** clinical decision support systems, computer physician order entry, health care costs, laboratory management, randomized controlled trials

## Abstract

**Introduction:**

Most of clinical laboratories are not properly reimbursed for their activity related to clinical trials (CTs) conducted in their institutions due to a lack of measurement strategies. We implemented a specific computer physician order entry (CPOE) environment for CTs in order to facilitate ordering to providers and estimate the associated costs to be compared with the standard of care (SOC).

**Materials and methods:**

Four specific electronic formularies, restricted to two new virtual CTs clinical services (onco - CT and haemo - CT), were implemented in January 2015. For each clinical trial displayed in the panels there were several box-cells that contained several profiles based on the different phase of the trials. Tests included in the profiles were the tests required by protocol. Laboratory costs (€) *per* patient were compared between the CTs services and their regular outpatients clinical services (onco - Out and haemo - Out, considered the SOC) for three years.

**Results:**

Costs *per* patient were higher for CTs services and increased progressively each year (25%, 70% and 70% and 0.6%, 2.7% and 17% in 2015, 2016 and 2017 for Oncology and Haematology, respectively). Taking into account all these differences and the number of patients attending a total difference in expense of + 130,377.7 € for the period 2015-2017 was obtained between CTs and outpatients services.

**Conclusions:**

Strategies through CPOE systems based on restricted and specific profiles for CTs ordering are a promising tool that can improve laboratory associated costs estimation and provide robust evidence in reimbursement negotiation processes with CTs sponsors.

## Introduction

In current clinical practice, randomised clinical trials are considered the best option when it comes to evaluating new treatments against standard of care (SOC) or current best practice (*1*). In patients with cancer they play a pivotal role in improving their care not only from a therapeutic point of view but from a palliative perspective as well. Besides, their management has experienced an increase in their complexity and, more importantly, in their costs over and above SOC (*2*). Due to this fact, health care institutions and their investigators have adopted a more demanding attitude with the sponsors of clinical trials in terms of cost recovery. However, the attribution and recovery of those costs can be very challenging (*3*).

In clinical trial, service support costs represent the ancillary patient care costs related to the research itself, which would finish once the clinical trial stops, even supposing the patient care continues to be dispensed. In this element additional investigations like imaging and laboratory tests are included as long as they would not continue to be supplied when trial stopped but patient care service continued to be provided.

Spanish 2015 legislation regarding clinical trials regulation concluded that all those extraordinary costs must be reflected in a contract between promoters and healthcare institutions (*4*). This contract must state the initial clinical trial budget, considering not only indirect costs but extraordinary direct costs such those expenses that are not incorporated in the standard treatment or the local treatment that would have been provided to patients had the trial not been accessible. All those elements must be met by the clinical trial promoter or sponsor.

Nowadays, most of the clinical laboratories struggle to identify the amount of tests performed annually related to cancer clinical trials conducted in their institutions. Therefore they are not able to estimate those extraordinary laboratory costs and claim a proper reimbursement from sponsors. In fact, most of the time, there is an underlying acceptance, without enough evidence, that states that laboratory costs related to patients enrolled in clinical trials are pretty much similar to those costs in patients on standard treatments. This is the main reason put forward by some sponsor companies and health institutions to not reimburse properly these expenses to clinical laboratories. Sadly, the lack of imaginative and well-designed measurement strategies and more advanced computer technologies could be considered as the major cause for this unfavourable and unprofitable scenario.

Clinical laboratories have several options for managing test adequacy with informatics and decision support rules as one of the most puissant and enduring tools at their disposal (*5*). A computer physician order entry (CPOE) system is a software that allows clinicians to enter orders directly into computer. These systems not only automate the clinical ordering process but also incorporate several features such as decision support mechanisms that improve the quality of healthcare and final patient outcomes (*6*). In addition, CPOE technology could potentially help users to separate laboratory activity related to routine healthcare from the activity linked to big research studies or randomized clinical trials. By means of customized electronic formularies or templates, clinical trial´s monitoring requests based upon treatment cycles or phases might be configured and displayed as clinical profiles (*7*). Afterwards all these data could be retrieved from Laboratory Information Systems (LIS) to feedback or, more importantly, collect economical information associated with this specific activity (*8*).

Here we describe the implementation at our Health Department of specific CPOE panels for laboratory tests related to clinical trials for two main purposes: firstly, to provide to our allowed users a friendlier and handier interface for test ordering; and secondly, to identify and quantify these requests and estimate the associated costs *per* patient. With the aim of discerning if those costs were higher than standard patients, we compared them with the calculated costs of SOC for oncologic and haematological outpatients. The difference (if existing) would be considered as the economical amount to be reimbursed to the laboratory by clinical trials promoters.

## Materials and methods

The study was conducted in University Clinic Hospital in Valencia (Spain), more specifically in its Biochemistry and Molecular Pathology Laboratory. In 2011, a CPOE system directly linked to our LIS, Gestlab (Cointec Ingenieros, Inc.) was implemented.

**Figure 1:**
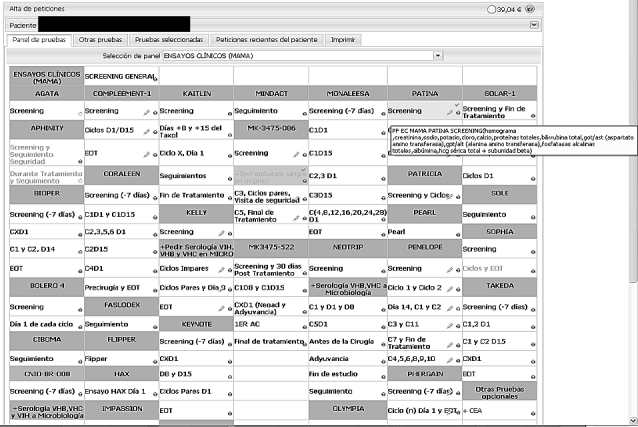
Panel for Breast Clinical Trials. Clinical trials are displayed in columns. Each coloured box-cell represents a different clinical trial profile heading (*e.g.* MONALEESA, PATINA, SOLAR-1). The rest of the box-cells represent profiles based on the phase of the trial (*e.g.* Screening, C1D1, EOT). Tests included in a profile are shown in a pop-up window when the cursor is hovered over the cell. When a specific profile is selected, its cost is displayed in euros in the upper right corner.

Specific electronic panels for clinical trials (CTs) ordering were implemented in January 2015. We designed three oncological panels (“Breast Trials”, “Lung Trials” and “Phase I/Miscellany Trials”) and one haematological panel (“Haematology Trials”) that were restricted to two new virtual clinical services created called “Oncology CTs” and “Haematology CTs” (OncCTs and HemCTs respectively). These new services contained all the same allowed users than the existing “Oncology” and “Haematology” outpatients services (OncOut and HemOut respectively). In these four panels (based in a “cell - box format”) all the active trials were displayed alphabetically. For each clinical trial there were several cells that contained the profiles based on the different phase of the trials (*e.g.* screening, cycles phase, end-of-treatment) (Figure 1). Tests included in the profiles were the tests required by protocol and they were always provided in advance by clinical trial´s data managers to the Clinical Biochemist responsible for LIS maintenance prior to their implementation. Once the clinical trial was finished, data managers had the obligation to inform Laboratory in order to cancel the trial (and its related profiles) and update the panel. While they were ordering, allowed physicians selected the desired profile based on the patient´s stage in the clinical trial, just ticking the respective box. Clinical trial´s profiles could only be ordered if the selected clinical service was “Oncology CTs” or “Haematology CTs”, otherwise clinical trials panels and their profiles were not available.

We collected retrospectively, from January 2015 to December 2017, the following data for the involved clinical outpatient services (OncOut, HemOut, OncCTs and HemCTs) *per* year: the number of total requests performed, the number of total patients attended, the number of clinical trials profiles ordered, and the total laboratory test cost in euros (€) according to Valencia´s Autonomic Health Tax Law. Additionally the ratios number of requests *per* patient (req/pat); number of ordered CTs profiles *per* request (pro/req); cost in € *per* request (cost/req) and cost in € *per* patient (cost/pat) were calculated. All the data were retrieved from our LIS. Clinical trials authorised during this three year period as well as the number of profiles ordered were classified based on their electronic panels.

All the OncOut and HemOut indicators could be considered, for comparison purposes, a SOC or the standard laboratory tests that would have been locally performed in the absence of clinical trials.

Data were analysed using SPSS 17.0 (SPSS Inc., Chicago, USA). In order to make statistical comparisons between the cost *per* patient of clinical trial and outpatient services *per* year, Student´s T-test was used considering a P value < 0.05 as statistical significant value.

## Results

Customised panels for clinical trials ordering were released 1^st^ of January 2015 for the oncology and haematology services involved (Figure 1). They were rapidly used by allowed providers without any complaints. A total number of 178 active clinical trials were displayed in our CPOE between 2015 and 2017 with Breast clinical trials (N = 53) and Phase I clinical trials (N = 48) being the most relevant (Table 1). Total of 7974 test profiles related to clinical trials were ordered in three years. From that number 70% of test profiles belonged to Breast and Phase I clinical trials (Table 1).

**Table 1:** Clinical trials displayed in our computer physician order entry between 2015 and 2017

**Type of CT**	**Active CTs,** **N (%)**	**CTs profiles ordered, N (%)**
Breast	53 (30)	3223 (40)
Phase I	48 (27)	2351 (30)
Haematology	37 (21)	1220 (15)
Miscellaneous	24 (13)	572 (7)
Lung	16 (9)	608 (8)
**TOTAL**	**178**	**7974**
CTs - clinical trials.		

Results for Oncologic services are summarised in Table 2. The ratio of requests *per* patients was higher for OncCTs than OncOut. Cost *per* patient was also higher and statistically significant for OncCTs *vs.* OncOut (P < 0.05) and increased progressively each year for OncCTs as well as the difference between OncCTs and OncOut (Table 4). Similar results were found for Haematologic services (Table 3) in terms of requests *per* patients but cost *per* patient was much more expensive than Oncologic services. Although cost *per* patient was always higher for HemCTs service, the differences in cost *per* patient between HemCTs and HemOut services were not significant except in 2017 (P < 0.05). Taking into account all these differences in cost per patient and the number of patients attended by CTs services each year, a total difference in expense of 130,377.7 € was obtained between CTs and outpatients services for the period 2015-2017 (Table 4).

**Table 2:** Results for Oncologic services for the period 2015 - 2017 displayed in computer physician order entry

**Clinical service and year**	**Requests performed**	**Patients attended**	**Requests *per* patient**	**CTs profiles ordered**	**CTs profiles *per* request**	**Laboratory cost (€)**	**Cost *per* request (€)**	**P-value for cost *per* request Out *vs.* CTs**	**Cost *per* patient (€)**	**P-value for cost *per* patient Out *vs*. CTs**
**Onc Out 2015**	23,780	17,769	1.3	-	-	1,047,773	44.1	0.680	59.0	< 0.001
**Onc CTs 2015**	1832	1013	1.8	1510	0.82	75,049	41.0		74.1	
**Onc Out 2016**	21,640	15,711	1.4	-	-	763,735	35.3	0.017	48.6	< 0.001
**Onc CTs 2016**	2633	1527	1.7	2522	0.96	126,209	47.9		82.7	
**Onc Out 2017**	21,230	16,251	1.3	-	-	794,193	37.4	0.060	48.9	< 0.001
**Onc CTs 2017**	2883	1597	1.8	2741	0.95	132,668	46.0		83.1	
“Cost *per* request” and “cost *per* patient” for outpatients *versus* clinical trials services have been compared using Student´s T-test. A P value < 0.05 was considered as statistical significant value. CT - clinical trial. Onc Out - Oncology Outpatients service. Onc CTs - Oncology Clinical Trials service.										

**Table 4:** Cost comparison between CTs and Outpatients services for the 2015 - 2017 period

**Year**	**Cost *per* patient CTs service (€)**	**Cost *per* patient Outpatient service (€)**	**Cost *per* patient difference (and %)** **(CTs - Out) (€)**	**P value**	**Patients** **attended in CT services**	**Total cost difference *per* year (€)**
**2015 Oncology**	74.1	59.0	+ 15.1 (+ 25.6)	< 0.001	1013	15,296.3
**2015 Haematology**	169.2	168.3	+ 0.9 (+ 0.6)	0.120	177	159.3
**2016 Oncology**	82.7	48.6	+ 34.1 (+ 70)	< 0.001	1527	52,070.7
**2016 Haematology**	158.1	153.9	+ 4.2 (+ 2.7)	0.082	245	1029.0
**2017 Oncology**	83.1	48.9	+ 34.2 (+ 70)	< 0.001	1597	54,617.4
**2017 Haematology**	177.2	151.0	+ 26.2 (+ 17.3)	< 0.001	275	7205.0
**TOTAL 2015 - 2017**					**4834**	**130,377.7**
“Cost *per* patient” for outpatients *versus* clinical trials services have been compared using Student´s T-test. A P value < 0.05 was considered as statistical significant value.						

**Table 3:** Results for Haematologic services for the period 2015 - 2017 displayed in computer physician order entry (CPOE)

**Clinical service and year**	**Requests performed**	**Patients** **attended**	**Requests *per* patient**	**CTs profiles ordered**	**CTs profiles *per* request**	**Laboratory cost (€)**	**Cost *per* request (€)**	**P-value for cost** ***per* request Out *vs*. CTs**	**Cost *per* patient (€)**	**P-value for cost *per* patient Out *vs.* CTs**
**Hem Out 2015**	8222	5717	1.4	-	-	961,947.0	117.0	< 0.001	168.3	0.120
**Hem CTs 2015**	391	177	2.2	323	0.83	30,000.1	76.7		169.2	
**Hem Out 2016**	11,700	8522	1.4	-	-	1,311,266.1	112.1	< 0.001	153.9	0.082
**Hem CTs 2016**	579	245	2.2	415	0.72	38,762.4	67.0		158.1	
**Hem Out 2017**	12,920	9439	1.4	-	-	1,425,380.5	110.3	< 0.001	151.0	< 0.001
**Hem CTs 2017**	599	275	2.2	463	0.77	48,666.6	81.3		177.2	
“Cost *per* request” and “cost *per* patient” for outpatients *versus* clinical trials services have been compared using Student´s T-test. A P value < 0.05 was considered as statistical significant value. CT - clinical trial. Hem Out - haematology outpatients service. Hem CTs - haematology clinical trials service.										

## Discussion

We implemented specific and restricted formularies for clinical trials ordering through a CPOE system. This fact brought two important advantages or key findings. On the one hand, we facilitated a handy, quick and useful setting for physicians, assuring complete adherence of CTs protocols proposed by their managers. On the other hand, our LIS could exploit from a statistical standpoint not only the volume of related profiles and tests but also the costs associated with this CTs activity. We have demonstrated in our study that laboratory costs *per* patient are higher for CTs patients if they are compared with the SOC or current best practice for an outpatient had the trials not been available. This is mainly due to a higher number of ordered requests for CTs patients *vs.* outpatients if we consider the same time period. Besides, for the oncologic CTs, we have also observed that cost *per* request difference increases gradually. As a general rule, a Laboratory may implement several strategies for request management in its CPOE (as we have developed last 5 years in our lab). Conversely, these interventions cannot be normally performed on CTs requests; otherwise they would violate promoter´s protocols. That´s why cost *per* request for an outpatient clinical service could decrease over time but not for CTs patients.

In our particular case for haematology clinical service, it´s more difficult to achieve a relevant cost *per* request reduction given that there are several compulsory tests of elevated cost performed in these patients that must be included in monitoring (*e.g.* genetic studies, bone marrow studies, flow cytometry panels, *etc.*). The decrease in cost *per* request observed for HemOut from 2015 to 2017, could be considered as modest and the differences between HemCTs and HemOut are minimal (in fact, they are only significant in 2017). We think that these findings could be explained if we take into account that most of the advanced tests described previously could have been ordered using HemOut as the allowed clinical service (and not HemCTs), so HemOut cost *per* patient could have been slightly overestimated while HemCTs cost *per* patient has been underestimated with the real difference between them higher than the obtained. Therefore, cost *per* patient difference between HemCT and HemOut could be a bit higher but unfortunately, and this is one of the limitations of our study, we cannot estimate such amount.

Even so, given that the cost for haematological CTs is marginal compared with the rest of oncological CTs, the main conclusions of our study remain unaffected: cost *per* patient in terms of laboratory investigations are much higher for CTs patients than for standard outpatients had the trials not been available and this difference should be reimbursed to clinical laboratories by clinical trial´s promoters. As far as we know, we are the first authors that describe and test a mechanism to estimate this expense which gives added value to our study.

Despite the lack of studies regarding this subject we find some examples that support our findings. In 2000, Evans *et al.* estimated that laboratory and imaging tests (which represented 17% and 39% of the total costs, respectively) were the major costs of conducting two phases II trials in lung cancer (*9*). They suggested that a potential reduction of the cost of clinical trials could be achieved if only the indispensable tests required by protocol were performed. In this same study they also solicited the opinion of 78 investigators regarding clinical trials funding. Surprisingly, investigators didn’t realize the importance of laboratory tests as one of the most representative costs of a phase II clinical trial. On the contrary, they regarded data management and nursing duties in chemotherapy delivery as the most important activities out of the SOC.

In 2013, Liniker *et al.* studied total costs related to CTs conducted at Addenbrooke´s Hospital (Liverpool, UK) over two years (*10*). On an average, a higher total cost saving was observed for commercial trials when compared with SOC, but when they reviewed the distribution of treatment costs incurred for an average patient, they also found an increase in blood tests cost for commercial trials (up to 400 £). Despite the total cost savings if all costs above SOC are expected to be fully reimbursed, it is reasonable to think that laboratories should be remunerated for this additional activity.

Finally, more recently, Tang *et al.* estimated the pathology cost avoidance for patients enrolled in phase III trials conducted by the NCIC Clinical Trials Group (*11*). Pathology cost avoidance (PCA) was defined by authors “when trial participation leads to provision of a pathology test so health care payers need not pay for it”. They identified four trials (three in colorectal cancer and one in breast cancer) that resulted in a total PCA of 4,194,849 $ for 1479 tests. Their estimates were conservative, given that they analysed only phase III trials and did not incorporate additional laboratory investigations that increment the costs related to CTs enrolment. The definition of PCA is a good example of service support costs, that is, the ancillary patient care costs related to the research itself, which would finish once the clinical trial had stopped, even supposing the patient care continued to be dispensed. As a concept PCA should be extended to all the laboratory costs not included in the SOC. Needless to say that, for sponsored trials, PCA reimbursement is a sponsor responsibility.

Even though clinical laboratory activity is regarded as indispensable in a correct patient management, its perception lamentably, as a relevant part in terms of cost of a clinical trial performance, is far from being significant or noticeable. Given that their associated costs are far from being insignificant and higher than current best practice, as we have demonstrated, we strongly recommend clinical laboratories to develop mechanisms in order to identify and quantify them and be properly remunerated.

In our particular case, since 2016, our laboratory receives from 4% of the total costs incurred by conducting a cancer clinical trial in our hospital as service support costs (internal estimation, data not shown). This economical amount in absolute terms allows us to hire on a yearly basis up to three people (laboratory technicians) in several areas as support staff. This reimbursement percentage is subject to change given that laboratory costs related to SOC are annually estimated.

Calculating the cost of laboratory tests or a test panel has always been a challenging task and it has been a widely accepted policy to estimate reimbursements due on the basis of charges, depending on payers involved (*12*). In publicly funded health care systems as Spanish National System, it is reasonable to expect industry to fund the costs related to the additional patient care costs as laboratory tests. We hope our study will encourage laboratories to identify these costs components of CTs and provide robust evidence in reimbursement negotiation processes with CTs sponsors.
